# Older Adults’ Acceptance of Technology During the Pandemic: The COVID Technology Acceptance Model (TAM)

**DOI:** 10.1093/geroni/igab046.3628

**Published:** 2021-12-17

**Authors:** Megan O'Connell, Kristen Haase, Allison Cammer, Shelley Peacock, Theodore Cosco, Lorraine Holtslander

**Affiliations:** 1 University of Saskatchewan, Saskatoon, Saskatchewan, Canada; 2 University of British Columbia, Vancouver, British Columbia, Canada; 3 Simon Fraser University, Vancouver, British Columbia, Canada

## Abstract

During the pandemic, technology-mediated communication was one of the few ways to maintain social and community connections. We explored how the pandemic impacted older adults’ use and appraisal of technology. In a random sample of 407 older adults (M age = 81.1 years; range 65-105 years) almost half (n = 161) reported they changed how they used technology to virtually connect with others during the pandemic, and 78 of these reported that this was new technology for them. We adapted the technology acceptance model (TAM) for the pandemic, the COVID-TAM, and describe how physical distancing led to new acceptance of technology due to an increased perception of usefulness of technology for maintaining community and social connections. The 71 older adults who denied using technology were asked about the reasons underlying their reluctance to use technology to access social networks and community events during the pandemic. Thematic analysis revealed factors consistent with a double-digital divide; lack of physical exposure to technology creates an additional psychological barrier to adoption of new technology. Of the technology-reluctant subgroup of older adults, few reported lack of perceived usefulness of technology during the pandemic. Instead, most reported lack of self-efficacy or fear of technology underlying their lack of technology use for social and community connections during the pandemic, which we incorporate into the COVID-TAM. Findings indicate that technology training can help mitigate this fear and increase social and community connections that are technology-mediated in circumstances where physical distancing is necessary.

